# Incorporating false negative tests in epidemiological models for SARS-CoV-2 transmission and reconciling with seroprevalence estimates

**DOI:** 10.1038/s41598-021-89127-1

**Published:** 2021-05-07

**Authors:** Rupam Bhattacharyya, Ritoban Kundu, Ritwik Bhaduri, Debashree Ray, Lauren J. Beesley, Maxwell Salvatore, Bhramar Mukherjee

**Affiliations:** 1 Department of Biostatistics, School of Public Health, University of Michigan, 1420 Washington Heights, Ann Arbor, MI 48109-2029 USA; 2 Indian Statistical Institute, Kolkata, West Bengal 700108 India; 3 Department of Epidemiology, Johns Hopkins University, Baltimore, MD 21205 USA; 4 Department of Biostatistics, Johns Hopkins University, Baltimore, MD 21205 USA; 5 Center for Precision Health Data Science, University of Michigan, Ann Arbor, MI 48109 USA

**Keywords:** Viral infection, Diagnostic markers, Viral infection

## Abstract

Susceptible-Exposed-Infected-Removed (SEIR)-type epidemiologic models, modeling unascertained infections latently, can predict unreported cases and deaths assuming perfect testing. We apply a method we developed to account for the high false negative rates of diagnostic RT-PCR tests for detecting an active SARS-CoV-2 infection in a classic SEIR model. The number of unascertained cases and false negatives being unobservable in a real study, population-based serosurveys can help validate model projections. Applying our method to training data from Delhi, India, during March 15–June 30, 2020, we estimate the underreporting factor for cases at 34–53 (deaths: 8–13) on July 10, 2020, largely consistent with the findings of the first round of serosurveys for Delhi (done during June 27–July 10, 2020) with an estimated 22.86% IgG antibody prevalence, yielding estimated underreporting factors of 30–42 for cases. Together, these imply approximately 96–98% cases in Delhi remained unreported (July 10, 2020). Updated calculations using training data during March 15-December 31, 2020 yield estimated underreporting factor for cases at 13–22 (deaths: 3–7) on January 23, 2021, which are again consistent with the latest (fifth) round of serosurveys for Delhi (done during January 15–23, 2021) with an estimated 56.13% IgG antibody prevalence, yielding an estimated range for the underreporting factor for cases at 17–21. Together, these updated estimates imply approximately 92–96% cases in Delhi remained unreported (January 23, 2021). Such model-based estimates, updated with latest data, provide a viable alternative to repeated resource-intensive serosurveys for tracking unreported cases and deaths and gauging the true extent of the pandemic.

## Introduction

COVID-19 was first diagnosed in Wuhan, China in December 2019 and was quickly declared a pandemic by the World Health Organization on March 11, 2020^1^. The first case in India was declared on January 30, and as of April 4, 2021, there have been 12,587,921 cases and 165,132 deaths reported^2^. India responded quickly, instituting a nationwide lockdown on March 25, when there were only 657 cases and 11 deaths^2,3^. Epidemiologic models can be used to monitor disease rates and inform public health interventions, but data quality will impact the ability of models to make accurate predictions. Underreporting of cases and deaths attributable to SARS-CoV-2 infection has hindered modeling efforts. This underreporting is primarily due to limited testing, deficiencies in the reporting infrastructure and a large number of asymptomatic infections.

Classical epidemiologic models, such as the Susceptible-Exposed-Infected-Removed (SEIR) compartmental model, have been used to predict the trajectory of the COVID-19 pandemic. For example, a modification of the standard SEIR model applied to Wuhan data and accounting for pre-symptomatic infectiousness, time-varying ascertainment rates, transmission rates and population identified that the outbreak had high covertness and high transmissibility^4^. This work estimated that 87% (with a lower bound of 53%) of the infections in Wuhan before March 8 were unascertained^4^. However, traditional SEIR models do not account for imperfect testing^5–7^. Individuals with a false negative diagnostic test will also remain unascertained and contribute to the compartment of latent unreported cases in a SEIR model.

**Figure 1:**
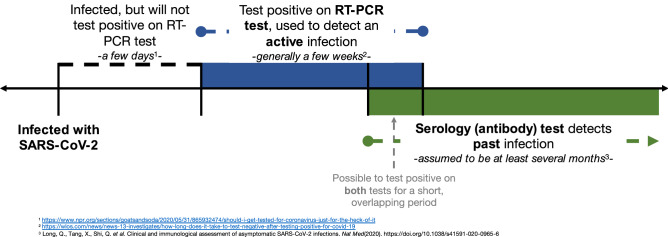
Timeline of COVID-19 diagnostic and antibody testing with respect to the infection and immune response time frame.

It is important to clarify that there are two classes of tests that are being discussed in the literature and are relevant to this paper: diagnostic tests and antibody tests. A diagnostic test (typically an RT-PCR test) is used to identify the presence of SARS-CoV-2, indicating an *active* infection^8^. An antibody test (i.e., a serology test) looks for the presence of antibodies, the body’s immune response to fight off SARS-CoV-2, indicating a *past* infection^9^. Figure 1 presents a timeline in terms of when these tests are administered during the course of an infection. Due to a large number of asymptomatic cases and limited number of tests, many infections do not get detected. Population-based seroprevalence surveys, therefore, give us an idea about the “true” number of infections including reported and unreported cases, and consequently, the ascertainment rate^10^. Thus, adjusted estimates of total number of cases and ascertainment rates based on serological surveys, when available, provide an option to validate model-based estimates of unreported cases and ascertainment rates. These estimates would usually be impossible to validate (except for in a simulation study) since these numbers are not observable in the real data.

Both diagnostic and antibody tests suffer from the issue of false negatives and false positives. For the RT-PCR test, a false negative is more worrisome since that means allowing an infected person a false safety assurance. In contrast, a false positive from an antibody test is of greater concern, since it gives the false impression that the person has been infected in the past, has gained some protection from the virus, and is unlikely to be infected again. The RT-PCR test is quoted to have a high false negative rate, ranging from 15 to 30% (i.e., low sensitivity, 85–70%), and a low false positive rate around 1–4% (i.e., high specificity, 99–96%)^11^. The antibody test assays are more precise—the commercial assays have sensitivity around 97.6% and specificity of 99.3% (DiaSorin) at about 15 days after infection^10^.

To address these data quality issues and the high rate of asymptomatic COVID-19 cases, we develop an extension to a standard SEIR model incorporating false negative rates in diagnostic testing to predict both the numbers of unreported cases and deaths and to estimate the rate at which COVID-19 cases and deaths are being underreported (unascertained). Our method segregates the traditional infected compartment into tested/untested and true positive/false negative compartments, thus accounting directly for misclassifications due to imperfections in the RT-PCR diagnostic tests. We apply this false negative-adjusted SEIR model to predict the transmission dynamics of SARS-CoV-2 in Delhi, the national capital region of India and one of the hotspots of COVID-19 in the country, using data from March 15 to June 30, 2020 for our original set of calculations and an updated range from March 15 to December 31, 2020 for another updated set of calculations. We make predictions across a range of possible sensitivities for the diagnostic test, all assuming perfect specificity.

To understand the true extent of spread of the novel coronavirus, the National Centre for Disease Control (NCDC) in India have performed five rounds of serological surveys in Delhi, among several such studies conducted across the world (Table 1)^12–44^. While limited on reported details, the first round of the Delhi Serology Study collected 21,387 random samples across 11 districts in Delhi between June 27 and July 10, 2020 and found COVID-19 antibodies present in 22.86% of samples^12,18^. This seroprevalence is the highest among the studies till July 2020 summarized in Table 1 but is similar to that found in New York City (22.70%), another large, densely populated area^42^. This indicates that Delhi, during July 2020, had high seroprevalence, even compared to worldwide epicenters and hotspots of COVID-19. The fifth and latest round of the Delhi serology study which collected 28,000 random samples across all the 272 municipal wards of Delhi between January 15–23, 2021 found an even higher seroprevalence, estimated at 56.13%^20^. This seroprevalence is the third highest among those summarized in Table 1, falling only behind the serosurvey in UK and that in Paris, France (among the working population)^31,39^. These numbers show that Delhi continued to have high seroprevalence among all the COVID-19 hotspots worldwide even in very recent times.

**Table 1:** Summary of COVID-19 seroprevalence studies across the world.

Location	Study design	Sample size	Estimated seroprevalence % (95% CI)	Reference
India	Pilot survey (April 2020) in 83 districts across 21 states	Unknown	0.73 overall 1.09 urban	*The Indian Express* (2020) url: https://indianexpress.com/article/explained/delhi-serological-survey-shows-antibodies-in-23-participants-what-does-this-mean-6516512/
India	1st national serosurvey (May–June 2020)—population-based survey of adults in representative samples from 700 villages/wards in 70 districts across 21 states conducted by ICMR	28,000	0.73 (0.34, 1.13)	Murhekar et al. (2020) *Indian Journal of Medical Research* https://doi.org/10.4103/ijmr.IJMR_3290_20
India	2nd national serosurvey (August–September 2020) of persons aged ≥ 10 years covering the same villages/wards/districts/states as previous serosurvey conducted by ICMR	29,082	6.6 (5.8, 7.4) overall 7.1 (6.2, 8.2) adults	Indian Council of Medical Research (2021) url: https://static.pib.gov.in/WriteReadData/userfiles/Modified%20ICMR_SecondSerosurvey_MMSP%20(1).pdf
India	3rd national serosurvey (December 2020-January 2021) of persons aged ≥ 10 years covering the same villages/wards/ districts/states as previous serosurveys conducted by ICMR; 100 healthcare workers per district	28,589 (general population) 7171 (healthcare workers)	21.4 adults 25.3 children aged 10–17 years 31.7 (28.1, 35.5) urban slum 26.2 (23.6, 28.8) urban non-slum 19.1 (18.0, 20.3) rural 25.7 (23.6, 27.8) healthcare workers	*BBC* (2021) url: https://www.bbc.com/news/world-asia-india-55945382
Chennai India	Household-based cross-sectional survey; participants selected from 51 wards from the city using multistage cluster sampling method	12,405	18.4 (14.8, 22.6) overall 16.3 (12.9, 20.3) male 20.3 (16.4, 25.0) female	Selvaraju et al. (2021) *Emerging Infectious Diseases* doi:10.3201/eid2702.203938
Delhi India	Prospective cross-sectional survey of healthcare workers in AIIMS hospital in the city	3739	13.0 *overall* 13.9 *male* 11.7 *female*	Gupta et al. (2020) *Indian Journal of Medical Research* https://doi.org/10.4103/ijmr.IJMR_3911_20
Delhi India	1st Delhi serosurvey (June–July 2020): randomly sampled individuals	21,387	22.9	*The Hindu* (2020) url: https://www.thehindu.com/news/cities/Delhi/percentage-of-people-with-antibodies-high/article32156162.ece
Delhi India	2nd Delhi serosurvey (August 2020) 3rd Delhi serosurvey (September 2020) 4th Delhi serosurvey (October 2020) Repeated, cross-sectional, multi-stage sampling design from all the 11 districts and 280 wards of the city-state, with two-stage allocation proportional to population size	15,046 17,049 15,015	28.4 (27.7, 29.1) 24.1 (23.4, 24.7) 24.7 (24.0, 25.4)	Sharma et al. (2020) *medRxiv* https://doi.org/10.1101/2020.12.13.20248123
Delhi India	5th Delhi serosurvey (January 2021): at least 100 participants from each of the 272 municipal wards of Delhi	28,000	56.1	*Hindustan Times* (2021) url: https://www.hindustantimes.com/cities/delhi-news/delhis-5th-sero-survey-over-56-people-have-antibodies-against-covid19-101612264534349.html
Karnataka India	Population-representative panel survey where households are randomly sampled to represent urban and rural areas of 5 state regions, and household members aged ≥ 12 years are chosen	1386	46.7 (43.3, 50.0) overall 44.1 (40.0, 48.2) rural 53.8 (48.4, 59.2) urban	Mohanan et al. (2021) *JAMA* https://doi.org/10.1001/jama.2021.0332
Kerala India	Repeated, cross-sectional, population-based survey of adults from 3 districts of this state	1193 (May 2020) 1281 (August 2020) 1246 (December 2020)	0.3 May 18–23 0.8 August 24–26 11.6 December 20–30	*Department of Health & Family Welfare, Government of Kerala* url: https://health.kerala.gov.in/pdf/Technical-paper-COVID-19-Sero-Surveillance-Round-3-ICMR.pdf
Mumbai India	Consent-based survey across three wards of this city with high COVID-19 growth and proximity to hotspots	6936 (out of 8800 invited)	40.5 overall 57.8 slum areas 16.0 non-slum areas	*The Indian Express* (2020) url: https://indianexpress.com/article/explained/mumbais-serosurvey-what-it-shows-about-gender-differences-in-infection-mortality-and-herd-immunity-6529186/
Pune India	Multi-stage cluster random sampling of participants recruited from 5 administrative sub-wards of this city selected randomly from 13 sub-wards classified as high incidence settings for a serosurvey	1659	51.3 (39.9, 62.4) overall 52.7 (41.7, 63.5) male 49.7 (37.5, 62.0) female	Ghose et al. (2020) *medRxiv* https://doi.org/10.1101/2020.11.17.20228155
Tamil Nadu India	Population-representative study conducted in all districts of this state with randomly selected participants (aged ≥ 18 years) in 888 clusters (comprising 30 participants in each cluster) during October–November 2020	26,135	31.6 (30.4, 32.8) overall 25.1 (24.2, 26.1) rural 36.7 (35.7, 37.7) urban 30.4 (29.6, 31.2) male 32.1 (31.1, 33.0) female	Malani et al. (2021) *medRxiv* https://doi.org/10.1101/2021.02.03.21250949
Brazil	1st national serosurvey (May 2020) 2nd national serosurvey (June 2020) Repeated cross-sectional study of one randomly selected person (≥ 1 year) per randomly selected household from 133 sentinel cities in all states	24,995 (1st ) 31,128 (2nd)	1st: 1.6 (1.4, 1.8) 2nd: 2.8 (2.5, 3.1)	Hallal et al. (2020) *Lancet Global Health* https://doi.org/10.1016/S2214-109X(20)30387-9
Hubei and Guangdong Provinces China	Cohort and location-specific surveys (Healthcare workers and their relatives, hospital outpatients, factory workers, hotel staff)	6919 (hospital settings) 10,449 (community settings)	3.8 (2.6, 5.4) healthcare workers, Wuhan 3.8 (2.2, 6.3) HOTEL staff members, Wuhan 3.2 (1.6, 6.4) family members, Wuhan	Xu et al. (2020) *Nature Medicine* https://doi.org/10.1038/s41591-020-0949-6
England	Series of consecutive weekly geographically representative sample across England (healthy adult blood donors, supplied by the National Health Service Blood and Transplant)	7000 (7 regions with 1000 per region)	14.8 London, week 18 3.5 North East, week 16 5.3 North West, week 16	Sero-surveillance of COVID-19 (2020) url: https://assets.publishing.service.gov.uk/government/uploads/system/uploads/attachment_data/file/888254/COVID19_Epidemiological_Summary_w22_Final.pdf
England	Personalized invitation-based survey of a random sample of adults from the National Health Service patient list	105,651	5.6 (5.4, 5.7) overall, unadjusted 6.0 (5.8, 6.1) overall, adjusted for test characteristics & weighted by population weights	Ward et al. (2021) *Nature Communications* https://doi.org/10.1038/s41467-021-21237-w
France	Repeated cross-sectional random sample of residual sera between March & May 2020 from two of the largest centralizing laboratories in France covering all regions	11,021	0.41 (0.05, 0.88) March 9–15 4.14 (3.31, 4.99) April 6–12 4.93 (4.02, 5.89) May 11–17	Le Vu et al. (2020) *medRxiv* https://doi.org/10.1101/2020.10.20.20213116
Paris France	Cross-sectional study of randomly sampled adults from sites with medical services in the city (food distribution sites, workers’ residences, emergency shelters) selected based on survey feasibility between March & June 2020	818	52.0 overall 28.0 (21.2, 35.5) food distribution site 89.0 (81.8, 93.2) workers’ residence 50.0 (46.3, 54.7) emergency shelter	Roederer et al. (2021) *Lancet Public Health* https://doi.org/10.1016/S2468-2667(21)00001-3
Essen Germany	Prospective cross-sectional monocentric study recruiting healthcare workers from University Hospital Essen	316	1.6	Korth et al. (2020) *Journal of Clinical Virology* https://doi.org/10.1016/j.jcv.2020.104437
Iran	Population-based cross-sectional study with randomly selected participants from the general population and a high-risk population across 18 cities in 17 Iranian provinces	3530 (general population) 5372 (high-risk population)	17.1 (14.6, 19.5) general population 20.0 (18.5, 21.7) high-risk population	Poustchi et al. (2021) *Lancet Infectious Diseases* https://doi.org/10.1016/S1473-3099(20)30858-6
Guilan Province Iran	Population-based cluster random sampling through phone call invitations	552 (196 households)	0.22 (0.19, 0.26) unadjusted 0.33 (0.28, 0.39) adjusted for imperfect testing 0.21 (0.14, 0.29) adjusted by population weight*s*	Shakiba et al. (2020) *medRxiv* https://doi.org/10.1101/2020.04.26.20079244
Kobe City Japan	Cross-sectional study on hospital outpatients	1000	3.3 (2.3, 4.6)	Doi et al. (2020) *medRxiv* https://doi.org/10.1101/2020.04.26.20079822
Spain	Two-stage random sampling of households stratified by province and municipality size	61,075 (point-of-care test) 51,958 (immunoassay) 35,883 (households)	5.0 (4.7, 5.4) point-of-care test 4.6 (4.3, 5.0) immunoassay	Pollán et al. (2020) *The Lancet* https://doi.org/10.1016/S0140-6736(20)31483-5
Sweden (9 regions)	Consecutive weekly region-specific surveys	1200 (per week)	7.3 Stockholm 4.2 Skåne 3.7 Västra Götaland	Public Health Agency Sweden (2020) url: https://www.folkhalsomyndigheten.se/nyheter-och-press/nyhetsarkiv/2020/maj/forsta-resultaten-fran-pagaende-undersokning-av-antikroppar-for-covid-19-virus/
Geneva Switzerland	Series of 5 consecutive weekly serosurveys among randomly selected participants from a previous population-representative survey, and their household members aged 5 years and older	2766 (1339 households; 341, 469, 577, 604 and 775 samples respectively in weeks 1–5.)	4.8 (2.4, 8.0) week 1 8.5 (5.9, 11.4) week 2 10.9 (7.9, 14.4) week 3 6.6 (4.3, 9.4) week 4 10.8 (8.2, 13.9) week 5	Stringhini et al. (2020) *The Lancet* https://doi.org/10.1016/S0140-6736(20)31304-0
UK	Cross-sectional study of randomly selected households from strictly-Orthodox Jewish community	1242 (343 households)	64.3 (61.6, 67.0) overall 68.8 (64.9, 72.5) men 59.7 (55.8, 63.5) women	Gaskell et al. (2021) *medRxiv* https://doi.org/10.1101/2021.02.01.21250839
USA	Cross-sectional study of respondents of all ages from 4 regional and 1 nationwide seroprevalence surveys, and community serosurvey data from randomly selected members of the general population	95,768	14.3 (IQR: 11.6, 18.5)	Angulo et al. (2021) *JAMA Network Open* https://doi.org/10.1001/jamanetworkopen.2020.33706
LA County, California USA	Invited enrollment, based on demographic match and geographical proximity to the testing centers	863 (out of 1952 invited)	4.06 (2.84, 5.60) unadjusted 4.34 (2.76, 6.07) adjusted for imperfect testing	Sood et al. (2020) *JAMA* https://doi.org/10.1001/jama.2020.8279
New York State USA	Convenience sampling of New Yorkers attending 99 grocery stores across 26 counties, containing 87.3% of the state's population, located all across the state	15,101	14.0 (13.3, 14.7) overall 22.7 (21.5, 24.0) New York City	Rosenberg et al. (2020) *Annals of Epidemiology* https://doi.org/10.1016/j.annepidem.2020.06.004
San Francisco Bay Area USA	Cohort-based recruitment of non-COVID patients and blood donors	387 (non-COVID patients) 1000 (blood donors)	0.26 (0.00, 0.76) non-COVID patients 0.10 (0.00, 0.56) blood donors	Ng et al. (2020) *medRxiv* https://doi.org/10.1101/2020.05.19.20107482
Santa Clara County, California USA	Ad-based recruitment, matched on geographic location and demographics	3330	1.5 (1.1, 2.0) unadjusted 1.2 (0.7, 1.8) adjusted for imperfect testing 2.8 (1.3, 4.7) adjusted for county demographic*s*	Bendavid et al. (2020) *medRxiv* https://doi.org/10.1101/2020.04.14.20062463

A simple proportional estimate based on these reported seroprevalences would tell us that Delhi, with approximately 19.8 million people, had around 4.6 million cumulative cases by July 10, 2020, and around 11.1 million cumulative cases by January 23, 2021. These numbers contrast sharply with the 109,140 cumulative cases (3,300 total deaths) reported in Delhi as of July 10, 2020 and the 633,739 cumulative cases (10,799 total deaths) as of January 23, 2021, which represent, respectively, approximately 0.55% and 3.20% of Delhi’s population. This disparity suggests that only about 2.4% of cases were being detected (underreporting factor of about 42) as of July 10, 2020, and as of January 23, 2021, that percentage has improved to only 5.7% (underreporting factor of about 18). The seroprevalence estimate also implies that the infection fatality rate (IFR) for Delhi was of the order of 0.07% or 717 per million as of July 10, 2020, which updates to 0.10% as of January 23, 2021. This IFR seems low compared to estimates worldwide^45^ and as such it may be reasonable to argue that some COVID-related deaths may also be unreported, or the cause of death misclassified. Uncertainty regarding the reporting of death data is further supported by the very small fraction of deaths in India that are medically reported^46^ and that the IFR estimates for SARS-CoV-2 from other studies in the world^45^ appear to be higher than influenza (as of 2018–19, infection fatality rate of influenza is at 961 per million or around 0.1%)^47^.

The availability of several rounds of seroprevalence estimates from the Delhi serology study provides a unique opportunity to validate model-predicted rates of latent unreported infections for our proposed false negative-adjusted SEIR model. The ELISA assay used in the Delhi serosurvey is a customized assay, some discussions on the development and imperfections of which are available both in recent literature and public media domain^48,49^. Based on these known imperfections, we perform adjustments of the reported case counts/infection estimates under different sensitivity and specificity assumptions for both the diagnostic and antibody (Ab) tests and compare the model-based estimates for the extent of underreporting to those obtained from the seroprevalence-based calculations. Other derived metrics such as case fatality rates and infection fatality rates are also presented. We use reported COVID-19 case and death count data from covid19india.org^2^. This framework can be adapted and applied to any set of reported case-counts where imperfect and limited testing exists.

## Results

### Extended SEIR model adjusted for misclassification

**Figure 2:**
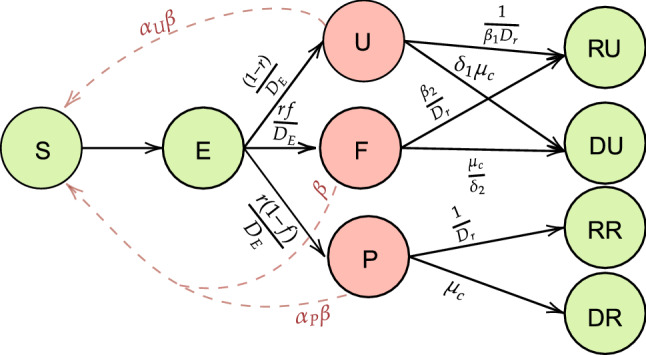
Diagram describing model compartments and transmissions for the extended SEIR model. For the detailed descriptions of the compartments and parameters, please refer to Supplementary Table 2 and the “Methods” section.

Figure 2 provides a schematic diagram of the proposed false-negative adjusted SEIR model. Under low (0.7), medium (0.85), meta-analyzed (0.952)^50^ and perfect (1) sensitivity, and perfect (1) specificity assumptions for the RT-PCR diagnostic test, we predict the total (reported and unreported) cases and deaths for Delhi using the proposed extended SEIR model.

**Figure 3:**
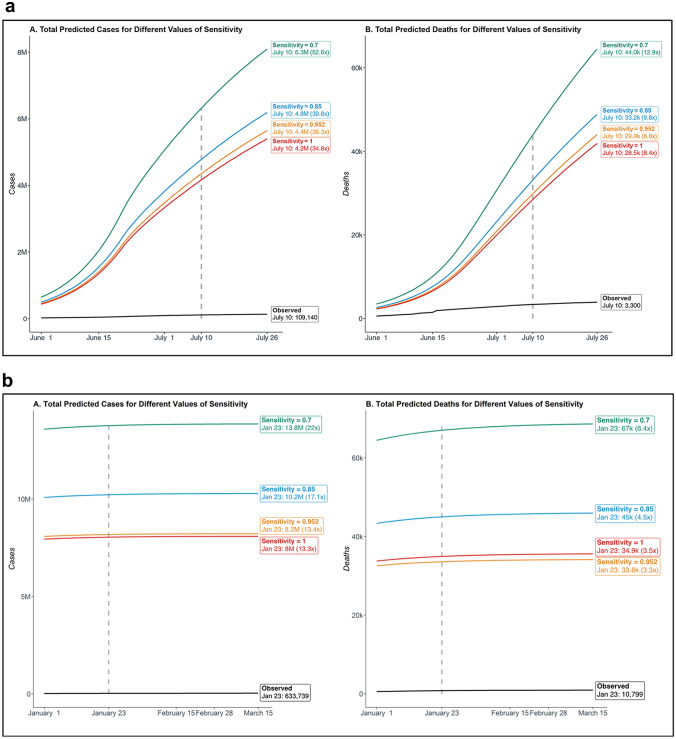
Summary of cumulative total (reported and unreported) cases and deaths for four different assumed values of sensitivity for the diagnostic RT-PCR test: 0.7, 0.85, 0.952, 1. In each subfigure, panels A and B respectively summarize the cases and deaths, along with their reported observed counterparts. The specificity of the diagnostic test is assumed to be 1. **(a)** Projections based on training data during March 15 to June 30, 2020, and testing period between June 1 to July 26, 2020. **(b)** Projections based on training data during March 15 to December 31, 2020, and testing period between January 1 to March 15, 2021.

Using data through June 30, 2020, this model estimates 4.8 million cases and 33,165 deaths on July 10, 2020 if we assume the RT-PCR test has a sensitivity of 0.85, and those predicted counts become 4.2 million and 28,499, respectively, if the sensitivity is assumed to be 1.0 (Fig. 3a). In contrast, the observed case and death counts are 109,140 and 3,300 reported in Delhi as of July 10, 2020^2^. Examining the ratio of predicted total number of cases and the predicted number of reported cases on July 10, 2020, the estimated case underreporting is within the range of 34–53 and the same quantity for underreported deaths is between 8 and 13 (Supplementary Table 1). According to this model, 97–98% of Delhi’s cases remain undetected as of July 10, 2020. The model predictions under the different scenarios considered and the results relative to daily reported/total case and death counts are summarized in Supplementary Figs. 1a and 2a.

Using data through December 31, 2020, this model estimates 10.2 million cases and 45,004 deaths on January 23, 2021 if we assume the RT-PCR test has a sensitivity of 0.85, and those predicted counts become 8.0 million and 34,949, respectively, if the sensitivity is assumed to be 1.0 (Fig. 3b). In contrast, the observed case and death counts are 633,739 and 10,799 reported in Delhi as of January 23, 2021^2^. Examining the ratio of predicted total number of cases and the predicted number of reported cases on January 23, 2021, the estimated case underreporting is within the range of 13–22 and the same quantity for underreported deaths is between 3 and 7 (Supplementary Table 1). According to this model, 92–96% of Delhi’s cases remain undetected as of January 23, 2021. The model predictions under the different scenarios considered and the results relative to daily reported/total case and death counts are summarized in Supplementary Figs. 1b and 2b.

### Future projections and variation of the underreporting factor through the course of the pandemic

We extended our projections of unreported case counts and the underreporting factors prospectively. Our projections for August 15, 2020 predict between 6.5 and 9.6 million cumulative (reported and unreported) cases in Delhi (across low to high false negative rate scenarios for the diagnostic test) (Supplementary Table 1). This provides us with a range of 35–54 for the underreporting factors for cases and a range of 8–13 for underreporting of deaths on August 15, 2020 (Supplementary Table 1). The temporal changes in the daily estimated case underreporting factors throughout the course of the pandemic is another crucial feature captured by our projections, as can be seen in Supplementary Fig. 3a. For the low (0.7) sensitivity scenario, the estimated case underreporting factor is 34 on June 1, 2020, the beginning of the first unlock period. This increases to 49 for June 20, 2020, when the daily number of tests and reported cases both increased.

The updated set of projections for February 15, 2021 predict between 8.1 and 13.8 million cumulative (reported and unreported) cases in Delhi (across low to high false negative rate scenarios for the diagnostic test) (Supplementary Table 1). This provides us with a range of 13–22 for the underreporting factors for cases and a range of 3–7 for underreporting of deaths on February 15, 2021 (Supplementary Table 1). Notably, our projections indicate that the underreporting factor for total cases is approximately constant over the period of January 1 to March 15, 2021, as can be seen in Supplementary Fig. 3b. For the low (0.7) sensitivity scenario, the estimated case underreporting stays at 22 throughout this period, and for the perfect (1.0) sensitivity scenario, this number decreases to 13.

### Naïve corrections to reported test results using known misclassification rates for tests

Since the total (reported and unreported) number of cases and subsequently, the underreporting factor, are not part of the observed data and therefore our SEIR model estimates cannot directly be validated, we validate these estimates using the estimated number of true infections predicted by the serosurvey data. However, the antibody tests are also imperfect and as such we also correct the seroprevalence estimates for imperfect testing.

Using varying (low to perfect) sensitivities and specificities for the diagnostic and antibody tests, we estimate that the true case count in Delhi as of July 10, 2020, lies between 4.4 and 4.6 million, which represents 30 to 42 times the number of reported cases (Table 2a). *These estimates strongly agree with model-based findings as reported in the previous subsection*, indicating that 96–97% cases in Delhi were underreported. Our updated estimate for the true case count in Delhi as of January 23, 2021 lies between 11.1 and 11.9 million, representing 17 to 21 times the number of reported cases (Table 2b). *Again, these estimates are in agreement with the model-based estimates from the previous subsection*, indicating that 94–95% cases in Delhi remained unreported even as recently as January 23, 2021.

**Table 2:** Summary of corrected number of cases, estimated underreporting factor, case-fatality rate based on reported cases and infection-fatality rate across different testing scenarios. Population size of Delhi is obtained from https://censusindia.gov.in/, and the testing, infection, recovery and fatality data are extracted from https://covid19india.org/.

(a) Calculations based on observed data as of July 10, 2020, and the first round of serological survey in Delhi								
		Antibody test (past infection)						
Diagnostic test		Serology test						
RT-PCR		Specificity	Sensitivity	Specificity	Sensitivity	Specificity	Sensitivity	
Specificity	Sensitivity	1	1	0.993	0.976	0.970	0.920	
1	1	4,526,217		4,527,984		4,418,221		Est. # true cases
		109,140		109,140		109,140		Corrected # reported cases
		41.5x		41.5x		40.5x		URF
		0.0302		0.0302		0.0302		CFR
		0.0007 (0.0073)		0.0007 (0.0073)		0.0007 (0.0075)		IFR (10 × adj.)
0.990	0.952	4,526,217		4,527,984		4,418,221		Est. # true cases
		107,929		107,929		107,929		Corrected # reported cases
		42.0x		42.0x		41.0x		URF
		0.0306		0.0306		0.0306		CFR
		0.0007 (0.0073)		0.0007 (0.0073)		0.0007 (0.0075)		IFR (10 × adj.)
0.990	0.850	4,526,217		4,527,984		4,418,221		Est. # true cases
		121,034		121,034		121,034		Corrected # reported cases
		37.4x		37.4x		36.5x		URF
		0.0273		0.0273		0.0273		CFR
		0.0007 (0.0073)		0.0007 (0.0073)		0.0007 (0.0075)		IFR (10 × adj.)
0.990	0.700	4,526,217		4,527,984		4,418,221		Est. # true cases
		147,346		147,346		147,346		Corrected # reported cases
		30.7x		30.7x		30.0x		URF
		0.0224		0.0224		0.0224		CFR
		0.0007 (0.0073)		0.0007 (0.0073)		0.0007 (0.0075)		IFR (10 × adj.)

(b) Calculations based on observed data as of January 23, 2021, and the fifth round of serological survey in Delhi								
		Antibody test (past infection)						
Diagnostic test		Serology test						
RT-PCR		Specificity	Sensitivity	Specificity	Sensitivity	Specificity	Sensitivity	
Specificity	Sensitivity	1	1	0.993	0.976	0.970	0.920	
1	1	11,113,457		11,325,962		11,819,615		Est. # true cases
		633,739		633,739		633,739		Corrected # reported cases
		17.5x		17.9x		18.7x		URF
		0.0170		0.0170		0.0170		CFR
		0.0010 (0.0049)		0.0010 (0.0048)		0.0009 (0.0046)		IFR (5 × adj.)
0.990	0.952	11,113,457		11,325,962		11,819,615		Est. # true cases
		563,529		563,529		563,529		Corrected # reported cases
		19.7x		20.1x		21.0x		URF
		0.0191		0.0191		0.0191		CFR
		0.0010 (0.0049)		0.0010 (0.0048)		0.0009 (0.0046)		IFR (5 × adj.)
0.990	0.850	11,113,457		11,325,962		11,819,615		Est. # true cases
		631,958		631,958		631,958		Corrected # reported cases
		17.6x		17.9x		18.7x		URF
		0.0171		0.0171		0.0171		CFR
		0.0010 (0.0049)		0.0010 (0.0048)		0.0009 (0.0046)		IFR (5 × adj.)
0.990	0.700	11,113,457		11,325,962		11,819,615		Est. # true cases
		769,340		769,340		769,340		Corrected # reported cases
		14.4x		14.7x		15.4x		URF
		0.0140		0.0140		0.0140		CFR
		0.0010 (0.0049)		0.0010 (0.0048)		0.0009 (0.0046)		IFR (5 × adj.)

(a) The URF is the ratio of the estimated number of true cases and the corrected number of reported cases. For the IFR, we report the estimate if we adjusted for 10 × death underreporting (10 × adj.).

(b) The URF is the ratio of the estimated number of true cases and the corrected number of reported cases. For the IFR, we report the estimate if we adjusted for 5 × death underreporting (5 × adj.).

*adj.* adjusted, *CFR* case-fatality rate, *est.* estimated, *IFR* infection-fatality rate, *URF* underreporting factor.

### Case fatality rate (CFR) and infection fatality rate (IFR)

The sensitivity and specificity of the diagnostic test impact our estimate of the case-fatality rate ($\frac{\#deaths}{\#reported cases}$), but not the infection-fatality rate ($\frac{\#deaths}{\#true infections}$). We estimate that the CFR lies between 2.24–3.06% as of July 10, 2020 (Table 2a), and between 1.40 and 1.91% as of January 23, 2021 (Table 2b). On the other hand, the sensitivity and specificity of the antibody test impact our estimates of the IFR. We estimate that the IFR lies between 0.07 and 0.08% based on the reported death counts as of July 10, 2020, and between 0.09 and 0.10% based on that as of January 23, 2021 (Table 2).

If we consider the hypothetical scenario of tenfold underreporting of deaths, as suggested by the SEIR model outputs (a range of 8–13), the infection-fatality rate estimate increases to 0.7–0.8% for July 10, 2020 (Table 2a). The updated SEIR model outputs indicate a range of 3–7 for the underreporting factor for deaths, and assuming fivefold underreporting of deaths, the adjusted infection-fatality rate estimate lies between 0.4 and 0.5% for January 23, 2021 (Table 2b). We are not able to perform any validation for the estimated underreporting factor for deaths as we do not have estimates of true death rates or excess deaths.

## Discussion

We developed an extension of the standard SEIR compartmental model to adjust for imperfect diagnostic testing. Applying our model on publicly available case and death count data for Delhi, we estimated the underreporting factor for cases to be somewhere between 34 and 53 and that for deaths to be somewhere between 8 and 13 on July 10, 2020 (with updated estimated ranges of 13–22 and 3–7 respectively on January 23, 2021). We obtained adjusted estimates of the underreporting factor for cases using the seroprevalence study (30–42 on July 10, 2020 and 17–21 on January 23, 2021), which largely agreed with those estimated from the model. Further, the estimated underreporting factors were seen to be more stable over an extended period of time with the new set of training data and testing period compared to the original calculations. Having an accurate idea about the underreporting factor and the extent of spread is extremely helpful in terms of tracking the growth of the pandemic and determining intervention policies. Since repeated serological surveys to track the ever-evolving seroconversion scenario are rarely viable options due to high expense in terms of cost, resources, and time, model estimates updated regularly with new incoming data provide an opportunity to monitor the underreporting factor and unreported cases and deaths.

### Limitations

(1) Our SEIR model incorporates only false negatives of the diagnostic tests but not false positives. We are more concerned about false negatives as this gives a false sense of safety to a patient and may increase the likelihood the person will engage in activities that will spread the disease. In addition, the false positive rates are quite low for PCR tests^11^. (2) We have refrained from incorporating a time-varying recovery rate in our model for several reasons. First, recovery data from India is not quite accurate and there is often a “catch up” period. The definition of recovery (e.g., negative COVID test, no symptoms) is also variable. As such, this may induce more noise. Second, modeling recoveries better change our estimate for “active” cases but does not affect what we consider in this paper, cumulative cases reported up to a give date. Third, including more time-varying parameters in the model will complicate the model further, and depending on the availability and quality of the recovery data, it may yield unstable/questionable fits. Finally, without directly considering the recovery rate to be time-varying, it is possible to effectively capture changes in the recovery rate by modifying one of the other parameters affecting recovery rate, like the mortality rate on which we have more data. For instance, one further generalized version of our model offers an option for time-varying mortality rate which has the potential to capture time-varying recovery^51^. (3) We used the seroprevalence estimate as a parallel, independent way of validating our model findings. An alternative approach for using serosurvey data is to introduce quarantine and immune compartments in the model structure and assume that symptomatic individuals are identified and successfully isolated with a given average delay from the onset of symptoms and that recovered individuals are never susceptible to an infection again^52^. We have not compared our method with this approach. (4) The implications of any such model-based adjustments depend heavily upon the reliability of the reported seroprevalence information. To that end, it is important to mention that many pertinent details were not released publicly in the first and fifth (latest) phases of the Delhi NCDC serology survey, such as the response and positivity rates stratified by age, sex, job type, district; sampling design and so on. A single reported number for the seroprevalence (22.86% and 53.16% respectively for the 1st and the 5th Delhi serosurveys) without sufficient detail on the survey design and assay used has limited use. (5) We do not know if individuals with antibodies are protected from re-infection, how long this protection lasts, the antibody levels needed to protect us from re-infections^53^, or whether a person with the antibody can still be contagious or show severe symptoms. The positive news from our estimates is that a large number of people in Delhi had the infection without feeling severe symptoms or needing clinical care.

## Conclusion

There have been debates about the path towards achieving herd immunity in India. The estimated range for the herd immunity threshold lies within 44–73% (based on worldwide estimated basic reproduction number of 1.8–3.8)^54,55^. For Delhi, and possibly even more so for other parts of India, herd immunity seems to be attainable as of recent dates but is certainly not a panacea we can rely on. Even based on the IFR obtained without adjusting for potential death underreporting and trusting the reported death counts as of January 23, 2021 (Table 2), if 50% of the 1.38 billion people in India get infected (a concept that many proponents of herd immunity have suggested), this would imply an estimated 690,000 deaths. This estimate skyrockets to a staggering 3.0–3.5 million deaths if we believe the current estimated underreporting factor for death from our proposed model. Although we could not validate the estimated underreporting factor for death, the quality of the reported death data is questionable. For example, a mid-2020 study attempting to model COVID-19 fatalities stratified by age-groups indicates that at least 1500–2500 deaths in Delhi in the 60 + age group have not been reported^56^. The high estimate of fatalities when adjusted for underreporting, along with these evidence for underreporting of deaths in India, calls for cautious actions, as India is beginning to see a second wave of the pandemic as recently as the beginning of April 2021^57^. Strong policy decisions directed towards containment of the new surge in infections and logistically efficient vaccination strategies are the need of the hour in this regard.

The appearance and spread of COVID-19 have taken the entire world by a storm, but a large number of examples from all across the world clearly depict that we can change the narrative and course of this virus through extensive testing, contact tracing, use of masks, hand hygiene and social distancing. For example, Delhi has seen tremendous success in turning the corner of the virus curve, with the time-varying reproduction number staying below unity for the larger part of the period between September 2020 and February 2021 (Supplementary Figs. 4–5). This trend of improved containment, however, seems to have reversed in the recent times, with the estimated time-varying reproduction number undergoing an alarming increase above unity during March–April 2021 (Supplementary Fig. 5). Several factors including public complacency, waning immunity that was acquired from past infections and the emergence of new variances may have contributed to this surge^58^. The appearance of these escalated numbers also calls for closer inspections of the serosurvey-based estimates, since a $>50\%$ seroprevalence and a spike in the number of new infections are theoretical antipodes in the context of a pandemic. Multiple potential reasons behind emerging biases in serosurvey estimates including non-representative sampling and assay characteristics have been discussed in recent literature, alongside possible ways of adjusting for such bias^59,60^.

Rapid and significant scientific advancements in both clinical and public health interventions have been made over the past year^61^. Data-driven policy decisions are crucial at this juncture. Our analytical framework for integrating diagnostic testing imperfections in the context of estimating unreported cases provides an alternative to conducting frequent serosurveys in Delhi. Validation of epidemiological model outputs against seroprevalence estimates inspires confidence in our inference and will hopefully prove to be a useful strategy for other case-studies.

## Methods

### Extended SEIR model adjusted for misclassification

We developed an extension of a standard SEIR model. In this model, the susceptible individuals (S) become exposed (E) when they are infected. After a latency period, exposed individuals are able to infect other susceptible individuals and are either untested (U) with probability $r$ or tested (T) with probability $1-r$. Tested individuals enter either the false negative compartment (F) with probability $f$ or the (true) positive compartment (P) with probability $1-f$. Individuals who are in the untested and the false negative compartments are considered unreported COVID-19 cases and enter either the recovered unreported (RU) or death unreported (DU) compartments. Similarly, those who tested positive move to either a recovered reported (RR) or death reported (DR) compartment. Figure 2 represents the SEIR model schematic, with arrows representing the possible transitions individuals in each compartment can undergo. The corresponding system of differential equations is presented below. The parameters and their initialization values used are described in Supplementary Table 2.

- $$\frac{\partial S}{\partial t}=-\beta \frac{S\left(t\right)}{N}\left({\alpha }_{P}P\left(t\right)+{\alpha }_{U}U\left(t\right)+ F\left(t\right)\right)+\lambda -\mu S\left(t\right).$$
- $$\frac{\partial E}{\partial t}=\beta \frac{S\left(t\right)}{N}\left({\alpha }_{P}P\left(t\right)+{\alpha }_{U}U\left(t\right)+F\left(t\right)\right)-\frac{E\left(t\right)}{{D}_{e}}-\mu E\left(t\right).$$
- $$\frac{\partial U}{\partial t}=\frac{(1-r)E(t)}{{D}_{e}}-\frac{U\left(t\right)}{{\beta }_{1}{D}_{r}}-{\delta }_{1}{\mu }_{c} U\left(t\right)-\mu U\left(t\right).$$
- $$\frac{\partial P}{\partial t}=\frac{r(1-f)E(t)}{{D}_{e}}-\frac{P\left(t\right)}{{D}_{r}}-{\mu }_{c}P\left(t\right)-\mu P\left(t\right).$$
- $$\frac{\partial F}{\partial t}=\frac{rfE(t)}{{D}_{e}}-\frac{{\beta }_{2}F\left(t\right)}{{D}_{r}}-\frac{{\mu }_{c} F\left(t\right)}{{\delta }_{2}}-\mu F\left(t\right).$$
- $$\frac{\partial RU}{\partial t}=\frac{U(t)}{{\beta }_{1}{D}_{r}}+\frac{{\beta }_{2}F(t)}{{D}_{r}}-\mu RU\left(t\right).$$
- $$\frac{\partial RR}{\partial t}=\frac{P\left(t\right)}{{D}_{r}}-\mu RR\left(t\right).$$
- $$\frac{\partial DU}{\partial t}={\delta }_{1}{\mu }_{c}U\left(t\right)+\frac{{\mu }_{c}F\left(t\right)}{{\delta }_{2}}.$$
- $$\frac{\partial DR}{\partial t}={\mu }_{c}P\left(t\right).$$

Here, $X(t)$ denotes the number of individuals in the compartment of interest $X$ at time $t$. Based on this set of differential equations, we calculate the basic reproduction number of the proposed model using the Next Generation Matrix Method^62^. The expression for ${R}_{0}$ turns out to be the following:

$${R}_{0}=\frac{\beta {S}_{0}}{\mu {D}_{e}+1}\left(\frac{{\alpha }_{u}\left(1-r\right)}{\frac{1}{{\beta }_{1}{D}_{r}}+{\delta }_{1}{\mu }_{c}+\mu }+\frac{{\alpha }_{p}r\left(1-f\right)}{\frac{1}{{D}_{r}}+{\mu }_{c}+\mu }+\frac{rf}{\frac{{\beta }_{2}}{{D}_{r}}+\frac{{\mu }_{c}}{{\delta }_{2}}+\mu } \right).$$

Here, ${S}_{0}=\frac{\lambda }{\mu }=1$, since we have assumed natural birth and death rate to be equal within this short period of time. In this setting, both $\beta$ and $r$ are time-varying parameters which are estimated using the Metropolis–Hastings MCMC method^63^. To estimate the parameters, we first need to be able to solve the differential equations, which is difficult to perform in this continuous-time setting. It is also worth noting that we do not require the values of the variables for each time point. Instead, we only need their values at discrete time steps, i.e., for each day. Thus, we approximate the above set of differential equations by a set of recurrence relations. For any compartment $X$, the instantaneous rate of change with respect to time $t$ (given by $\frac{\partial X}{\partial t}$) is approximated by the difference between the counts of that compartment on the ${\left(t+1\right)}^{th}$ day and the ${t}^{th}$ day, that is $X\left(t+1\right)-X(t)$. Starting with an initial value for each of the compartments on the Day 1 and using the discrete-time recurrence relations, we can then obtain the solutions of interest. Some examples of these discrete-time recurrence relations are presented below.

- $$E\left(t+1\right)-E\left(t\right)=\beta \frac{S\left(t\right)}{N}\left({\alpha }_{P}P\left(t\right)+{\alpha }_{U}U\left(t\right)+F\left(t\right)\right)-\frac{E\left(t\right)}{{D}_{e}}-\mu E\left(t\right),$$
- $$U\left(t+1\right)-U\left(t\right)=\frac{\left(1-r\right)E\left(t\right)}{{D}_{e}}-\frac{U\left(t\right)}{{\beta }_{1}{D}_{r}}-{\delta }_{1}{\mu }_{c} U\left(t\right)-\mu U\left(t\right),$$
- $$P\left(t+1\right)-P\left(t\right)=\frac{r\left(1-f\right)E\left(t\right)}{{D}_{e}}-\frac{P\left(t\right)}{{D}_{r}}-{\mu }_{c}P\left(t\right)-\mu P\left(t\right),$$
- $$F\left(t+1\right)-F\left(t\right)=\frac{rfE\left(t\right)}{{D}_{e}}-\frac{{\beta }_{2}F\left(t\right)}{{D}_{r}}-\frac{{\mu }_{c} F\left(t\right)}{{\delta }_{2}}-\mu F\left(t\right).$$

The rest of the differential equations can each be similarly approximated by a discrete-time recurrence relation. These parameters are estimated using training data from Delhi from March 15 to June 30, 2020 for our first set of analyses, and from March 15 to December 31, 2020 for our updated set of analyses. The training data were divided into multiple periods, in accordance with the lockdown and unlock procedures employed by the government of India, as described in Supplementary Table 3. Using these, we obtained predictions for dates ranging from June 1 through August 15, 2020, for the first set of analyses, and between January 1 to March 15, 2021 for the updated set of analyses. Since we used an MCMC algorithm to estimate the parameters and the posterior means of the compartment sizes, it is easy to obtain empirical posterior credible intervals based on the full set of MCMC draws to quantify the uncertainty associated with these estimates and projections. However, we deliberately refrained from reporting the uncertainty estimates in this paper to avoid intricacies in presentation of the results that may hinder the central message. Further, we assumed the RT-PCR test specificity to be 1 and did not incorporate false positives arising from the diagnostic test to avoid additional assumptions for model identifiability.

### Naïve corrections to reported test results using known misclassification rates

Notations: Let *N* = population size, *X* = number of true cases in the population (hence *N – X* = number of non-cases in the population), *T* = number of people tested, *S* = number of true cases tested (hence *T – S* = number of non-cases tested, X – *S* = number of true cases not tested, *N – X – T* + *S* = number of non-cases not tested), *P* = number of positive tests (also, therefore, cumulative number of reported cases, hence *T – P* = number of negative tests). Note that *X* and *S* are the only two unknowns in this setting. Also, let us assume that the sensitivity of the test of interest is $\boldsymbol{\alpha }$ and the specificity of the same is $\beta$. With that, we can set up the following equation, because there are two ways a test can be positive, as can be seen in Supplementary Fig. 6.

$$P=S\times \alpha +\left(T-S\right)\times \left(1-\beta \right)\Rightarrow \frac{P}{T}=\frac{S}{T}\times \alpha +\left(1-\frac{S}{T}\right)\times \left(1-\beta \right).$$

Adjusting the terms, we get the following expression for $S$.

$$S=T\times \frac{\frac{P}{T}+\beta -1}{\alpha +\beta -1}.$$

Assuming that the proportion of cases among those tested stays the same as the original population (random and hence homogenous testing), we can replace $S$ by $\frac{TX}{N}$, which will lead to the following updated equation.

$$\frac{P}{T}=\frac{X}{N}\times \alpha +\left(1-\frac{X}{N}\right)\times \left(1-\beta \right).$$

Solving this, we get the following expression for $X$.

$$X=N\times \frac{\frac{P}{T}+\beta -1}{\alpha +\beta -1}.$$

Thus, these two expressions give us, for a given set of $\alpha$ and $\beta$, the corrected number of reported cases ($S$), and also the estimated number of true (reported and unreported) cases ($X$). For the computation of $S$, we use $\frac{P}{T}=\frac{\mathrm{109,140}}{\mathrm{747,109}}\approx 0.146$, the test positive rate of the RT-PCR tests in Delhi as of July 10^2^. For the computation of $X$, we use $\frac{P}{T}=\frac{\mathrm{4,889}}{\mathrm{21,387}}\approx 0.229$, the positive rate reported by the first round of the Delhi serological survey^12–14^. For the updated analysis based on more recent data, these numbers are updated to $\frac{\mathrm{644,064}}{\mathrm{10,289,461}}\approx 0.062$ and $\frac{\mathrm{15,716}}{\mathrm{28,000}}\approx 0.561$ respectively. Once we get these estimates, we can compute the adjusted underreporting factor as $URF=\frac{X}{S}$. Also, assuming that $D$ denotes the cumulative number of deaths till a date of interest, we can compute the corrected versions of case fatality rate and infection fatality rate as $CFR=\frac{D}{S}$ and $IFR=\frac{D}{X}$, respectively. Further, if we want to adjust for a potential scenario where for every M death due to COVID-19, we observe 1 death (M-fold underreporting for deaths), we can update the IFR estimate as $IFR=\frac{MD}{X}$. We calculate our adjusted IFR estimates for $M=10$ for the July 10, 2020 computations, and for $M=5$ for the January 23, 2021 computations. Based on the data from Delhi, we use $D=3300$ for July 10, 2020, and $D=\mathrm{10,994}$ for January 23, 2021^2^. We also use a population size of $N=1.98\times {10}^{7}$ based on recent population data^64^, since the last official census in India was performed in 2011, and the number reported there may not be representative of the current scenario.

A critical question here is the choice of $\alpha$ and $\beta$ for the two tests to ensure our computations reflect adjustments made based on sensible and realistic scenarios. Based on previously reported sensitivity and specificity levels for the diagnostic test^10,49^, we used the combinations $\alpha =\beta =1 \left(\text{perfect test}\right), \alpha =0.952 {\text{and}} \beta =0.99$, $\alpha =0.85 {\text{and}} \beta =0.99$, ${\text{and}} \alpha =0.7 {\text{and}} \beta =0.99$. The serological assay used by NCDC is a customized assay, and we referred to existing literature on and publicly available discussions on this particular assay, alongside literature on serological assays in general^48,49^, and decided to use the combinations of $\alpha =\beta =1 \left(\text{perfect test}\right)$, $\alpha =0.976 {\text{and}} \beta =0.993$, ${\text{and}} \alpha =0.92 {\text{and}} \beta =0.97$.

## Supplementary Information

Supplementary Information.

## Data Availability

All data used in our analyses are available at http://covind19.org.

## Code availability

All our computational codes are available at http://covind19.org.
