# Author Correction: Incorporating false negative tests in epidemiological models for SARS-CoV-2 transmission and reconciling with seroprevalence estimates

**DOI:** 10.1038/s41598-021-96603-1

**Published:** 2021-08-20

**Authors:** Rupam Bhattacharyya, Ritoban Kundu, Ritwik Bhaduri, Debashree Ray, Lauren J. Beesley, Maxwell Salvatore, Bhramar Mukherjee

**Affiliations:** 1 Department of Biostatistics, School of Public Health, University of Michigan, 1420 Washington Heights, Ann Arbor, MI 48109‑2029 USA; 2 Indian Statistical Institute, Kolkata, West Bengal 700108 India; 3 Department of Epidemiology, Johns Hopkins University, Baltimore, MD 21205 USA; 4 Department of Biostatistics, Johns Hopkins University, Baltimore, MD 21205 USA; 5 Center for Precision Health Data Science, University of Michigan, Ann Arbor, MI 48109 USA

Correction to: *Scientific Reports* https://doi.org/10.1038/s41598-021-89127-1, published online 07 May 2021

The original version of this Article contained errors in Figure 2, where the colours within each circle and dashed line arrows were omitted.

The original Figure 2 and accompanying legend appear below.

**Figure 2:**
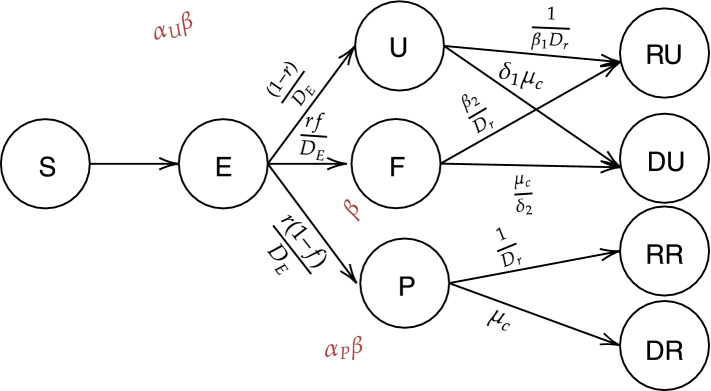
Diagram describing model compartments and transmissions for the extended SEIR model. For the detailed descriptions of the compartments and parameters, please refer to Supplementary Table 2 and the “Methods” section.

The original Article has been corrected.

