# Supplementary material for: Incorporating false negative tests in epidemiological models for SARS-CoV-2 transmission and reconciling with seroprevalence estimates
Source: Sci Rep. 2021 May 7;11:9748. doi: 10.1038/s41598-021-89127-1 (PMC8105357; doi:10.1038/s41598-021-89127-1)

**Supplementary Information for:**
**Incorporating false negative tests in epidemiological models for SARS-CoV-2 transmission and reconciling with seroprevalence estimates**

Rupam Bhattacharyya[1]; Ritoban Kundu[2]; Ritwik Bhaduri[2]; Debashree Ray[3,4]; Lauren J. Beesley[1,5]; Maxwell Salvatore[1,5]; Bhramar Mukherjee[1,5]*.

[1]: Department of Biostatistics, University of Michigan, Ann Arbor, MI 48109, USA
[2]: Indian Statistical Institute, Kolkata 700108, West Bengal, India
[3]: Department of Epidemiology, Johns Hopkins University, Baltimore, MD 21205, USA
[4]: Department of Biostatistics, Johns Hopkins University, Baltimore, MD 21205, USA
[5]: Center for Precision Health Data Science, University of Michigan, Ann Arbor, MI 48109, USA

*: Corresponding author. Address - Department of Biostatistics, School of Public Health, University of Michigan, 1420 Washington Heights, Ann Arbor, MI 48109-2029, USA. Telephone – (734) 764-6544. Email – bhramar@umich.edu.

## SUPPLEMENTARY TABLES

**Supplementary Table 1. Summary of extended SEIR model results for Delhi.** Predicted cumulative case and death counts and corresponding underreporting factors with respect to the observed data are presented for July 10, 2020, August 1, 2020, August 15, 2020 (based on training data from March 15-June 30, 2020 with a testing period between June 1-August 15, 2020), and for January 23, 2021, February 15, 2021, February 28, 2021 (based on training data from March 15-December 31, 2020 with a testing period between January 1-March 15, 2021). The specificity of the RT-PCR test is assumed to be 1.

| Sensitivity of RT-PCR Test | Predicted Reported Cases | Predicted Total Cases | Under-reporting Factor for Cases | Predicted Reported Deaths | Predicted Total Deaths | Under-reporting Factor for Deaths |
|---|---|---|---|---|---|---|
| | | | **July 10, 2020** | | | |
| **0.700** | 119,920 | 6,318,663 | 52.6 | 3,386 | 43,978 | 12.9 |
| **0.850** | 119,879 | 4,780,982 | 39.8 | 3,384 | 33,165 | 9.8 |
| **0.952** | 119,728 | 4,351,781 | 36.3 | 3,380 | 29,685 | 8.8 |
| **1.000** | 119,603 | 4,164,568 | 34.8 | 3,376 | 28,499 | 8.4 |
| | | | **August 1, 2020** | | | |
| **0.700** | 161,071 | 8,607,604 | 53.4 | 5,650 | 70,923 | 12.6 |
| **0.850** | 163,494 | 6,617,126 | 40.5 | 5,687 | 53,920 | 9.5 |
| **0.952** | 163,866 | 6,044,666 | 36.9 | 5,689 | 48,580 | 8.5 |
| **1.000** | 163,656 | 5,778,839 | 35.3 | 5,680 | 46,250 | 8.1 |
| | | | **August 15, 2020** | | | |
| **0.700** | 177,805 | 9,536,967 | 53.6 | 6,762 | 83,195 | 12.3 |
| **0.850** | 182,904 | 7,432,470 | 40.6 | 6,876 | 63,931 | 9.3 |
| **0.952** | 183,927 | 6,813,487 | 37.0 | 6,895 | 57,715 | 8.4 |
| **1.000** | 183,792 | 6,515,786 | 35.5 | 6,886 | 54,922 | 7.9 |
| | | | **January 23, 2021** | | | |
| **0.700** | 626,672 | 13,766,492 | 22.0 | 10,478 | 67,040 | 6.4 |
| **0.850** | 599,685 | 10,222,324 | 17.1 | 9,975 | 45,004 | 4.5 |
| **0.952** | 608,816 | 8,179,143 | 13.4 | 10,182 | 33,598 | 3.3 |
| **1.000** | 606,606 | 8,043,085 | 13.3 | 10,142 | 34,949 | 3.5 |
| | | | **February 15, 2021** | | | |
| **0.700** | 629,003 | 13,833,208 | 22.0 | 10,659 | 68,138 | 6.4 |
| **0.850** | 599,196 | 10,270,789 | 17.1 | 10,156 | 45,674 | 4.5 |
| **0.952** | 610,816 | 8,209,960 | 13.4 | 10,355 | 33,993 | 3.3 |
| **1.000** | 608,773 | 8,077,126 | 13.3 | 10,318 | 35,411 | 3.5 |
| | | | **February 28, 2021** | | | |
| **0.700** | 629,449 | 13,847,316 | 22.0 | 10,705 | 68,440 | 6.4 |
| **0.850** | 599,617 | 10,280,352 | 17.1 | 10,203 | 45,850 | 4.5 |
| **0.952** | 611,116 | 8,214,909 | 13.4 | 10,399 | 34,089 | 3.3 |
| **1.000** | 609,138 | 8,083,312 | 13.3 | 10,363 | 35,528 | 3.4 |

**Supplementary Table 2. Description of extended SEIR model parameters.**

| Parameter | Value | Description |
|---|---|---|
| $\beta$ | *Time-varying* | Rate of infectious transmission by infected, tested individuals with false negative results. |
| $\alpha_p$ | 0.5 | Ratio of rate of spread of infection by tested positive patients to that by false negatives. $\alpha_p < 1$ represents the scenario where individuals who test positive are infecting susceptible individuals are a lower rate than infected individuals with false negative test results. |
| $\alpha_u$ | 0.7 | Scaling factor for the rate of spread of infection by untested individuals. $\alpha_u$ is assumed to be < 1 as U mostly consists of asymptomatic or mildly symptomatic cases who are known to spread the disease at a much lower rate than those with higher levels of symptoms. |
| $D_e$ | 5.2 | Incubation period (in days). |
| $D_r$ | 17.8 | Mean number of days until recovery for infected individuals. |
| $D_t$ | 0 | Mean number of days for the test result to come after a person is tested. Under the assumption of instantaneous test results, this is taken to be zero. *This results in the E compartment being directly distributed into three compartments U, F and P, i.e., there remains no testing compartment.* |
| $\mu_c$ | 0.0562 | Death rate attributable to COVID-19 which is equivalent to inverse of the average number of days for death starting from the onset of disease times the probability of death of an infected individual. |
| $\lambda, \mu$ | $3.95 \times 10^{-5}$ | Natural birth and death rates (assumed to be equal). |
| $r$ | *Time-varying* | Probability of being tested for infectious individuals. |
| $f$ | 0.3, 0.15, 0.048, 0 | Probability of a false negative RT-PCR diagnostic test result. |
| $\beta_1, \frac{1}{\beta_2}$ | 0.6 ($\beta_1$) 0.7 ($\beta_2$) | Scaling factors for rate of recovery for undetected and false negative individuals respectively. Both $\beta_1$ and $\beta_2$ are assumed to be less than 1. It is assumed that the recovery rate is slower for the false negative individuals than the detected ones because they are not getting any hospital treatments. The condition of Untested individuals is not so severe as they consist of mostly asymptomatic people. So, they are assumed to recover faster than the Current Positive Ones. |
| $\delta_1, \frac{1}{\delta_2}$ | 0.3 ($\delta_1$) 0.7 ($\delta_2$) | Scaling factors for death rate for undetected and false negative individuals respectively. Both $\delta_1$ and $\delta_2$ are assumed to be less than 1. Same as before, the death rate for False Negative ones is assumed to be higher than the Current detected Positive as they are not receiving proper treatment. While, for the Untested ones, the death rate is taken to be lesser because they are mostly asymptomatic. So, their probability of dying is much less. |

**Supplementary Table 3. Training data periods according to interventions.** The periods marked in *italics* are only used for the updated set of analysis with training data up to December 31, 2021, and predictions during January-March 2021.

| | |
|---|---|
| Pre-lockdown | March 15 – March 24 |
| Lockdown 1.0 | March 25 – April 14 |
| Lockdown 2.0 | April 15 – May 3 |
| Lockdown 3.0 | May 4 – May 17 |
| Lockdown 4.0 | May 18 – May 31 |
| Unlock 1.0 | June 1 – June 30 (This period is divided into two parts: June 1 – June 19 and June 20 – June 30, since there was a high increase in the number of tests on June 20.) |
| *Unlock 2.0* | *July 1 – July 31* |
| *Unlock 3.0* | *August 1 – August 31* |
| *Unlock 4.0* | *September 1 – September 30* |
| *Unlock 5.0* | *October 1 – October 31* |
| *Unlock 6.0* | *November 1 – November 30* |
| *Unlock 7.0* | *December 1 – December 31* |

## SUPPLEMENTARY FIGURES

**Supplementary Figure 1. Bar plots of predicted reported and unreported daily cases.** Panels A, B, C and D depict the predictions under assumed sensitivity of the diagnostic test at 0.7, 0.85, 0.952 and 1, respectively. Panel E shows the consistency of the predictions with the observed data. The specificity of the diagnostic test is assumed to be 1.

***Supplementary Figure 1a. Projections based on training data from March 15 to June 30, 2020. Projections extend between June 1 to July 26, 2020.***

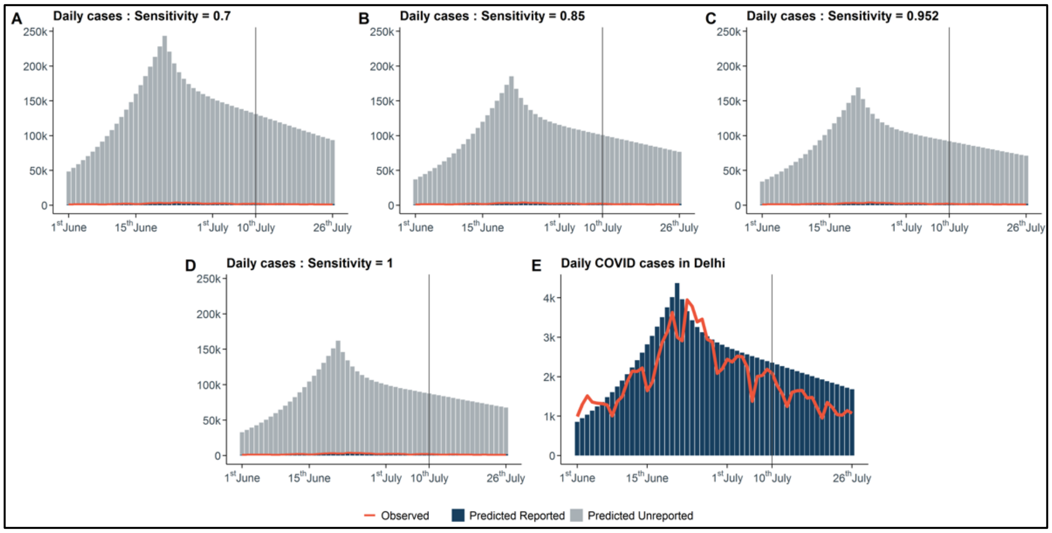


***Supplementary Figure 1b. Projections based on training data from March 15 to December 31, 2020. Projections extend between January 1 to March 15, 2021.***

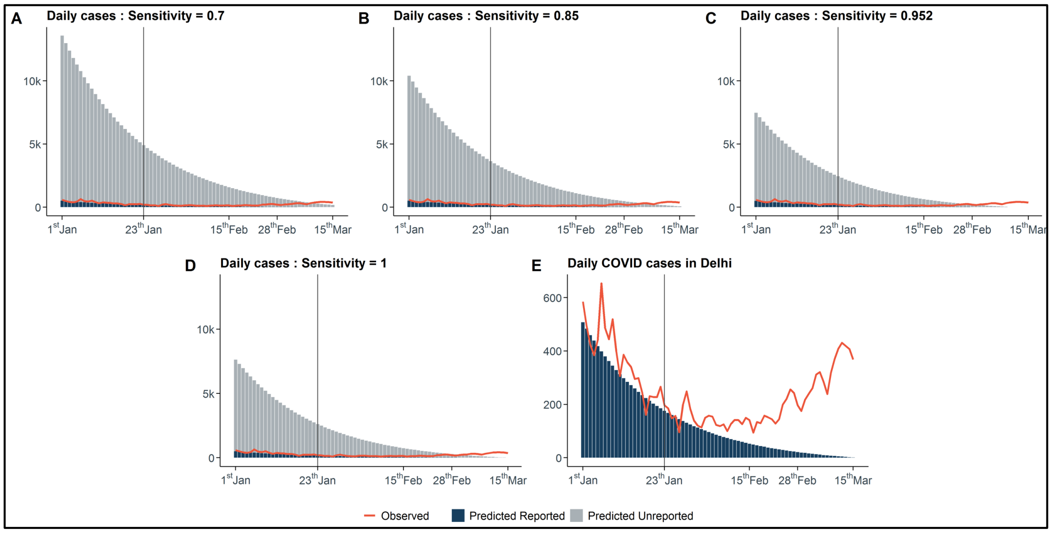

**Supplementary Figure 2. Bar plots of predicted reported and unreported daily deaths.** Panels A, B, C and D depict the predictions under assumed sensitivity of the diagnostic test at 0.7, 0.85, 0.952 and 1, respectively. Panel E shows the consistency of the predictions with the observed data. The specificity of the diagnostic test is assumed to be 1.

***Supplementary Figure 2a. Projections based on training data from March 15 to June 30, 2020. Projections extend between June 1 to July 26, 2020.***

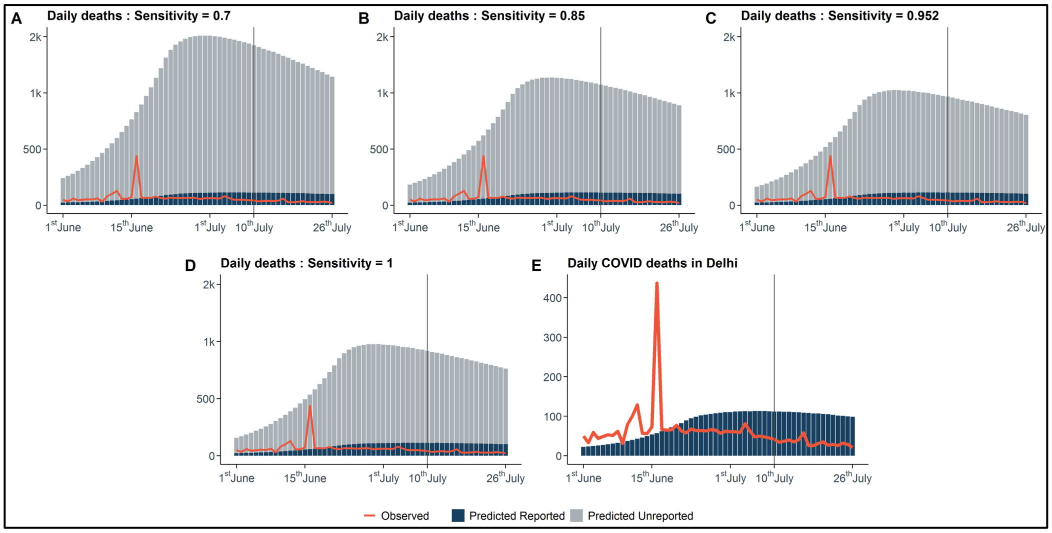


***Supplementary Figure 2b. Projections based on training data from March 15 up to December 31, 2020. Projections extend between January 1 to March 15, 2021.***

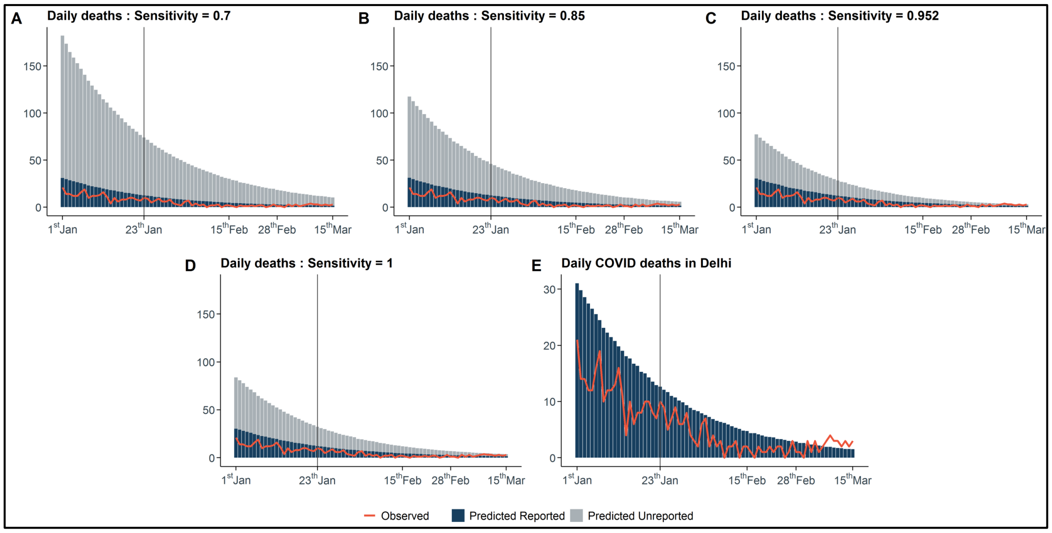

**Supplementary Figure 3. Plot of predicted underreporting factor for total cases** The four colored lines depict the predictions under assumed sensitivity of the diagnostic test at 0.7, 0.85, 0.952 and 1. The specificity of the diagnostic test is assumed to be 1.

***Supplementary Figure 3a. Projections based on training data from March 15 to June 30, 2020. Projections extend between June 1 to July 26, 2020.***

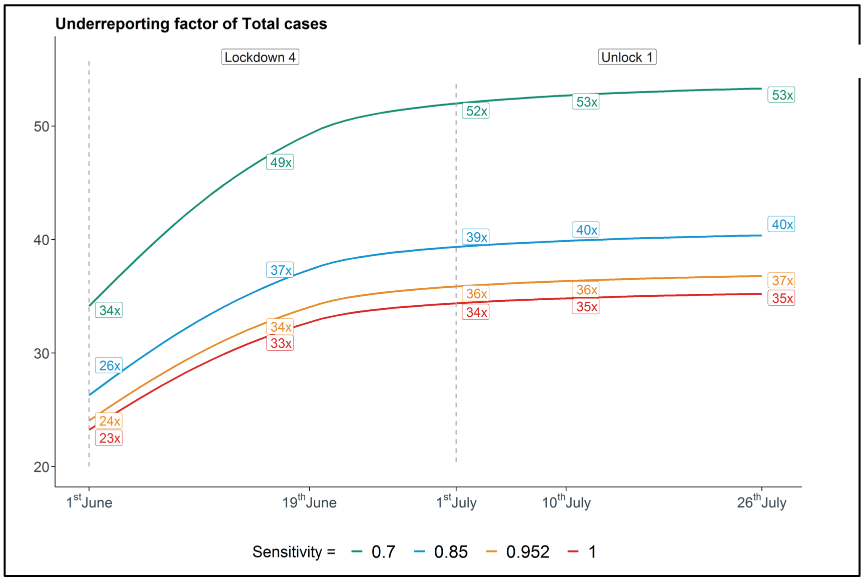


***Supplementary Figure 3b. Projections based on training data from March 15 up to December 31, 2020. Projections extend between January 1 to March 15, 2021.***

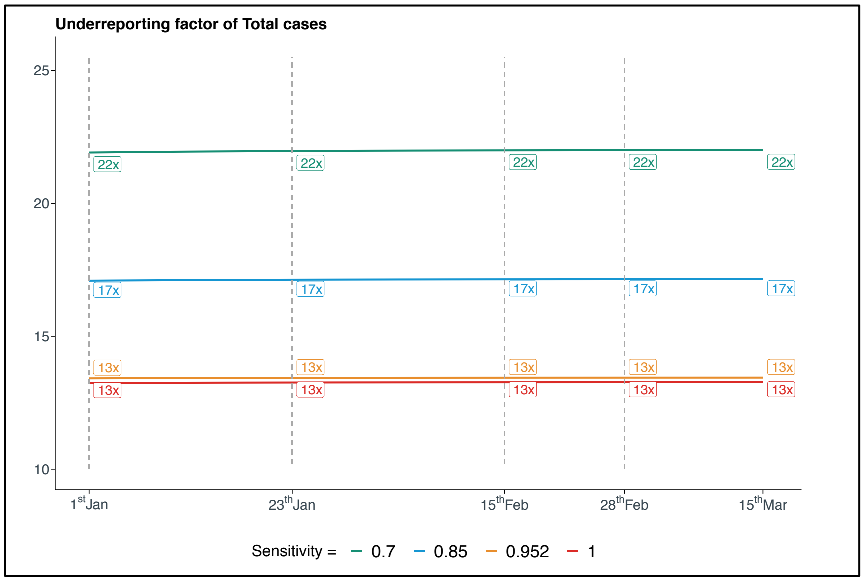

**Supplementary Figure 4. Observed daily COVID-19 case, recovery and fatality counts for Delhi during March 15, 2020 – March 15, 2021.**

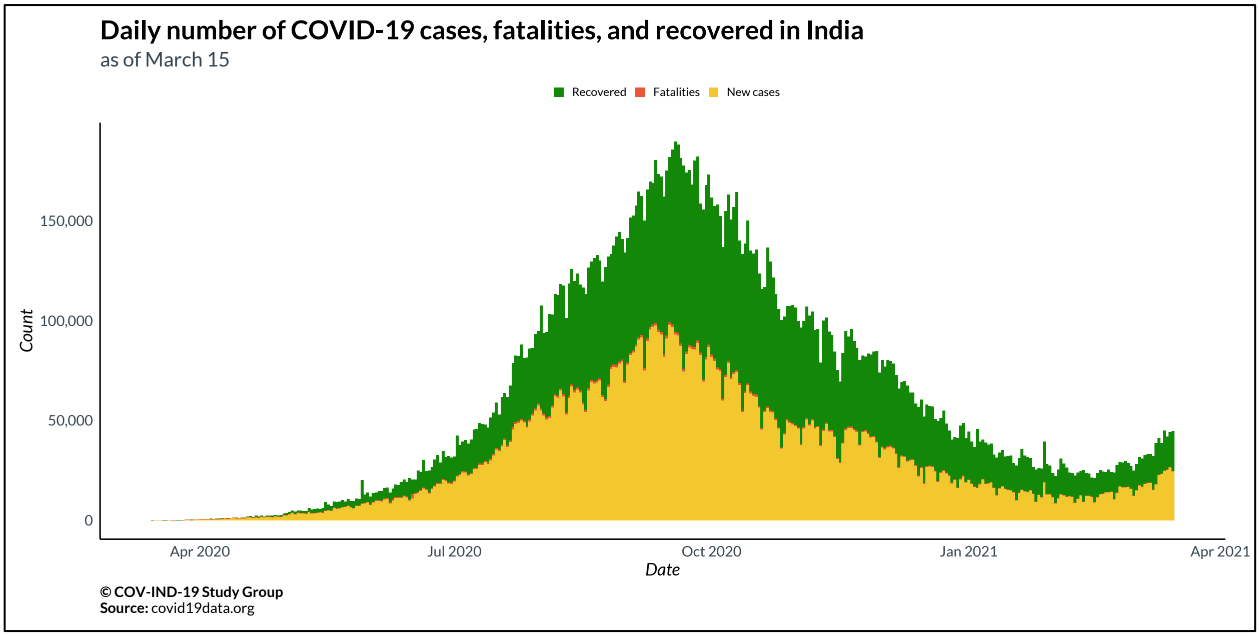


**Supplementary Figure 5. Estimated time-varying reproduction number, R (and 95% posterior credible interval), for COVID-19 in Delhi during March 15, 2020 to March 15, 2021.** Computations were performed using the R package EpiEstim.

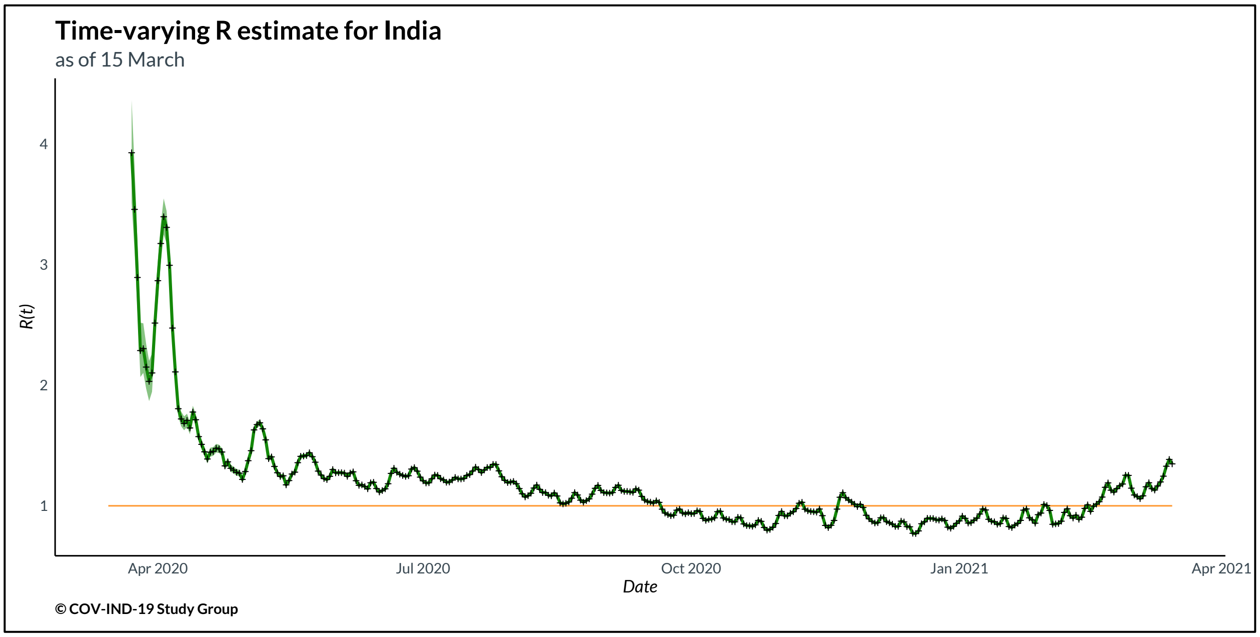

**Supplementary Figure 6. Diagram of testing decisions.** Dark lines indicate the break-up of the population in terms of true infection status; green and red lines indicate (correct and incorrect, respectively) decisions based on testing procedure. Here, we have referred to the diagnostic test, and hence, the active infection status. Similar framework applies to the antibody test and the past infection status.

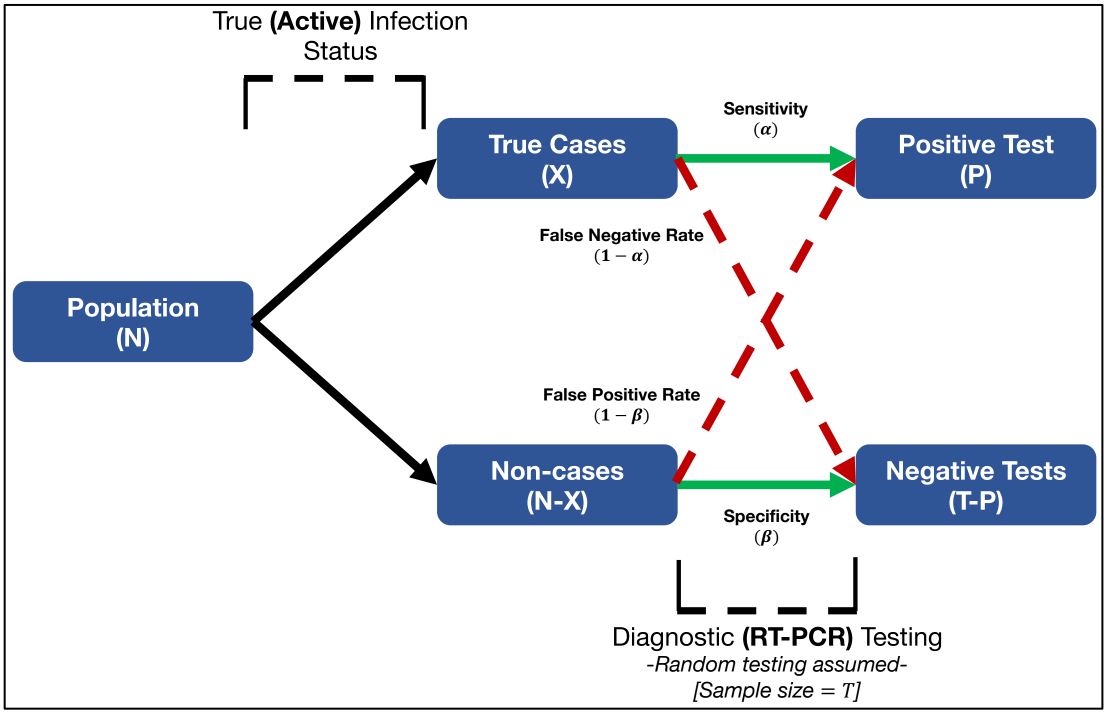

Supplement: Supplementary file 1 — Supplementary Information. [file 41598_2021_89127_MOESM1_ESM.pdf]
